# Structural basis of transcription-coupled RNA damage by incorporation of oxidized ribonucleotides

**DOI:** 10.1073/pnas.2602266123

**Published:** 2026-04-14

**Authors:** Peini Hou, Chanjoo Lee, Jenny Chong, Juntaek Oh, Dong Wang

**Affiliations:** ^a^Department of Pharmaceutical Sciences, Skaggs School of Pharmacy and Pharmaceutical Sciences, University of California, San Diego, CA 92093; ^b^Department of Regulatory Science, Graduate School, Kyung Hee University, Seoul 02447, Republic of Korea; ^c^Institute of Regulatory Innovation through Science, Kyung Hee University, Seoul 02447, Republic of Korea; ^d^Institute of Integrated Pharmaceutical Sciences, Kyung Hee University, Seoul 02447, Republic of Korea; ^e^Department of Cellular and Molecular Medicine, University of California, San Diego, CA 92093; ^f^Department of Chemistry and Biochemistry, University of California, San Diego, CA 92093

**Keywords:** RNA damage, transcription fidelity, structural biology, RNA polymerase II, oxidative damage

## Abstract

Oxidative stress induces damage to DNA, RNA, and nucleotide pools. Unlike well-studied DNA damage, the formation of RNA damage and the impact of an oxidized ribonucleotide pool on transcription fidelity are poorly understood. Here, we investigate the structural basis of transcription-coupled RNA damage and the effect of 8-oxo-guanosine triphosphate (8-oxo-rGTP) on RNA polymerase II (Pol II) transcription fidelity control steps. We revealed that the incorporation efficiency of 8-oxo-rGTP opposite a dC template is comparable to that of GTP. In contrast, the incorporation efficiency of 8-oxo-rGTP opposite a dA template is ~150-fold more efficient than that of GTP. For the extension step, Pol II extends substantially faster from a 3′-8-oxo-rG:dC base pair than from a 3′-8-oxo-rG:dA base pair. For the proofreading step, strikingly, Pol II EC with 3′-8-oxo-rG:dA base pair is much more resistant to backtracking and proofreading than Pol II EC with 3′-8-oxo-rG:dC base pair. Using X-ray crystallography, we revealed that 8-oxo-rGTP adopts different prechemistry binding sites depending on whether it is paired with a dC or a dA template. Upon incorporation, the nucleobase of 8-oxo-rG flips to the *syn*-conformation to form a Hoogsteen pair with a dA template, whereas it remains in the *anti*-conformation to form a Watson–Crick pair with a dC template. Collectively, our work demonstrates that nucleotide-pool oxidation can directly affect Pol II fidelity control steps and elongation dynamics and induce RNA damage in a transcription-coupled manner.

## Significance

Cells are continuously exposed to oxidative stress, which lead to oxidation damage on DNA, RNA as well as nucleotide pools. However, the formation of oxidative RNA damage and the impact of an oxidized ribonucleotide pool on transcription fidelity is poorly understood. Here, we investigate the structural basis of transcription-coupled RNA damage and effect of 8-oxo-guanosine triphosphate (8-oxo-rGTP) on RNA polymerase II (Pol II) transcription fidelity control steps. Our work reveals that nucleotide-pool oxidation can directly and greatly affect Pol II fidelity control and elongation dynamics and introduce RNA damage. This study reveals an understudied link between oxidative stress and transcriptional dysregulation, which has a broad implication in aging, neuronal diseases, and cancer.

**Fig. 1.**
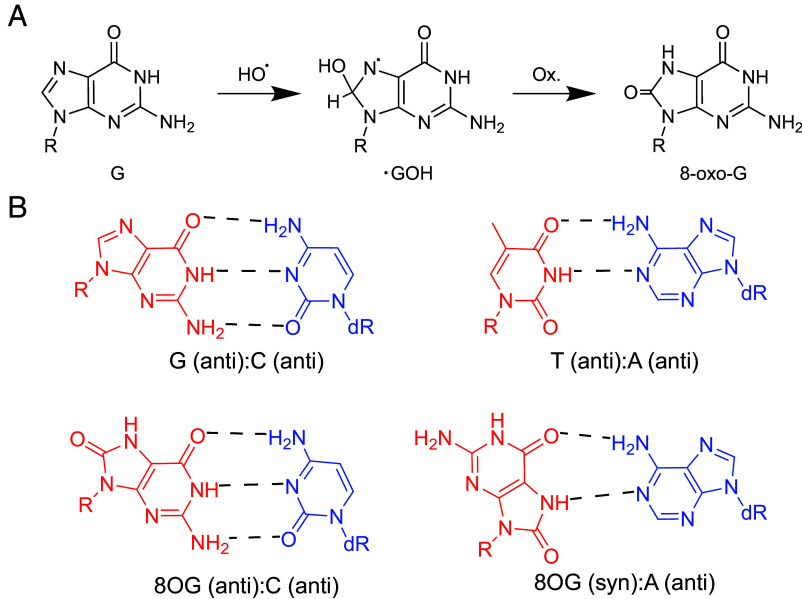
Formation of 8-oxo-7,8-dihydroguanine from guanine (G) and comparison of natural base pairing and 8-oxo-guanine base pairing. (*A*) 8-oxo-7,8-dihydroguanine is generated from reacting with oxygen at the C8 position. (*B*) 8-oxo-guanine pairs with cytosine (C) in anti-conformation and with adenine (A) in syn-conformation using Hoogsteen base pairing, while for natural base pairing, G is base paired with C and Thymine (T) is base paired with A using canonical Watson–Crick base pair.

Cells are continuously exposed to oxidative stress originating from endogenous metabolism and exogenous agents. Reactive oxygen species generated during respiration, ultraviolet radiation, and inflammation impose persistent pressure on the fidelity of genetic information storage and transfer (1–5). As one of the most abundant oxidative damage, 8-oxo-7,8-dihydroguanine (hereafter referred to as 8-oxoG; also abbreviated as 8OG) are present in both nucleic acids and nucleotide pools (Fig. 1*A*). A key feature of 8-oxoG is its conformational plasticity and dual coding potential. It forms a canonical Watson–Crick pair with cytosine in the *anti*-conformation and a Hoogsteen pair with adenine in the *syn*-conformation (Fig. 1*B*) (6–11). This dual coding potential of 8-oxoG underlies mutagenesis at both DNA and RNA level and has been linked to transcriptional dysregulation, aging, and cancer (12–15).

Most previous studies have been focused on the impact of 8-oxoG lesions in DNA template on transcription (6, 7, 16, 17). In particular, recent structural and kinetic studies have elucidated the detailed molecular basis of how Pol II deals with oxidized DNA lesions at template strand in a stepwise manner and found that the 8-oxoG DNA template behaves very differently in error-free and error-prone transcription (6, 7, 17). For error-free cytidine triphosphate (CTP) incorporation, the 8-oxoG template remains in anti-conformation during template loading, nucleotide binding, and incorporation steps. As for error-prone adenosine triphosphate (ATP) incorporation, 8-oxoG template initially adopts anti-conformation during template loading and the initial nucleotide binding step, and then adopts the syn-conformation, base-paired with newly incorporated adenosine monophosphate (AMP) at postchemistry state. Molecular dynamics (MD) simulations further revealed that the 8-oxoG template switches from an anti- to a syn-conformation by partially backtracking and subsequently reloading into the +1 site. Intriguingly, 8-oxoG:rA base pair can escape Pol II proofreading and Pol II is able to extend from the 8-oxoG:rA base pair. These findings established that the 8-oxoG DNA lesion can reconfigure the active site and reshape the transcription fidelity control and the lesion bypass of oxidative DNA damage.

8-oxoG lesions are also present in RNA as well as nucleotide pools. RNA oxidative damage can be introduced in a posttranscription (direct oxidation) or in a transcription-coupled manner via incorporation of oxidized nucleotide substrates. While Nudix hydrolases (MutT in bacteria and MTH1/NUDT1 in mammals) can recognize and hydrolyze 8-oxo-dGTP and 8-oxo-rGTP to their monophosphates, protecting genome and transcriptome fidelity (18, 19), the efficiency for 8-oxo-rGTP hydrolysis is around 50-fold less than that of 8-oxo-dGTP (20). Oxidative stress can substantially elevate levels of oxidized rNTPs up to 2 to 5% (21, 22), thereby increasing the likelihood of cellular polymerase incorporation of these oxidized rNTPs as substrates (23, 24). Recent structural work showed that 8-oxo-rGTP can be efficiently incorporated by DNA polymerase μ during double-strand break repair and captured ternary complexes with 8-oxo-rGTP paired with template dA, highlighting that oxidized ribonucleotide can serve as efficient substrates for a repair polymerase (25). However, how these oxidized ribonucleotide triphosphate (rNTPs) affect eukaryotic transcription process, in particular, how eukaryotic Pol II handles oxidized ribonucleotides in its active site during transcription remains poorly understood. Once incorporated within the transcriptome, these oxidized residues can significantly compromise the fidelity of the mRNA pool. Such RNA damage has been shown to perturb base pairing–dependent processes like pre-mRNA splicing, induce ribosome stalling, and promote translational errors, eventually leading to proteotoxic outcomes and neurodegeneration (26).

In this manuscript, we took a combined presteady state single turnover kinetics and structural biology approach to investigate the effects of 8-oxo-guanosine triphosphate (8-oxo-rGTP) on the Pol II transcription process. We focused on individual transcription fidelity checkpoints, including incorporation, extension, and proofreading steps. We found that Pol II can incorporate 8-oxo-rGTP opposite a dC or a dA template with distinct mechanisms. In the case of a dC template, the 8-oxo-rGTP substrate binds in the addition site (A-site) and forms a Watson–Crick 8-oxo-rG:dC pair in the anti-conformation which is stabilized by trigger loop (TL) closure. The incorporated 8-oxo-rG remains paired to a dC in a pretranslocation state that advances efficiently and is readily cleaved by transcription factor IIS (TFIIS)-stimulated cleavage. However, in the case of a dA template, the key event is the flipping of 8-oxo-rG to syn-conformation that creates a Hoogsteen base pair at +1 position upon reaction. This geometry introduces a hydrogen bond between the 8-oxo-rG and Rpb2 E529 in fork loop 2, which stabilizes a pretranslocation state. This newly formed contact inhibits both forward translocation and backtrack, therefore slows down lesion bypass and suppresses TFIIS-mediated cleavage. Taken together, our work reveals that nucleotide-pool oxidation can directly affect Pol II fidelity control and elongation dynamics and introduce transcription-coupled RNA damage.

## Results

### Incorporation Step: 8-Oxo-rGTP Is Efficiently Incorporated Opposite dA and dC Templates.

**Fig. 2.**
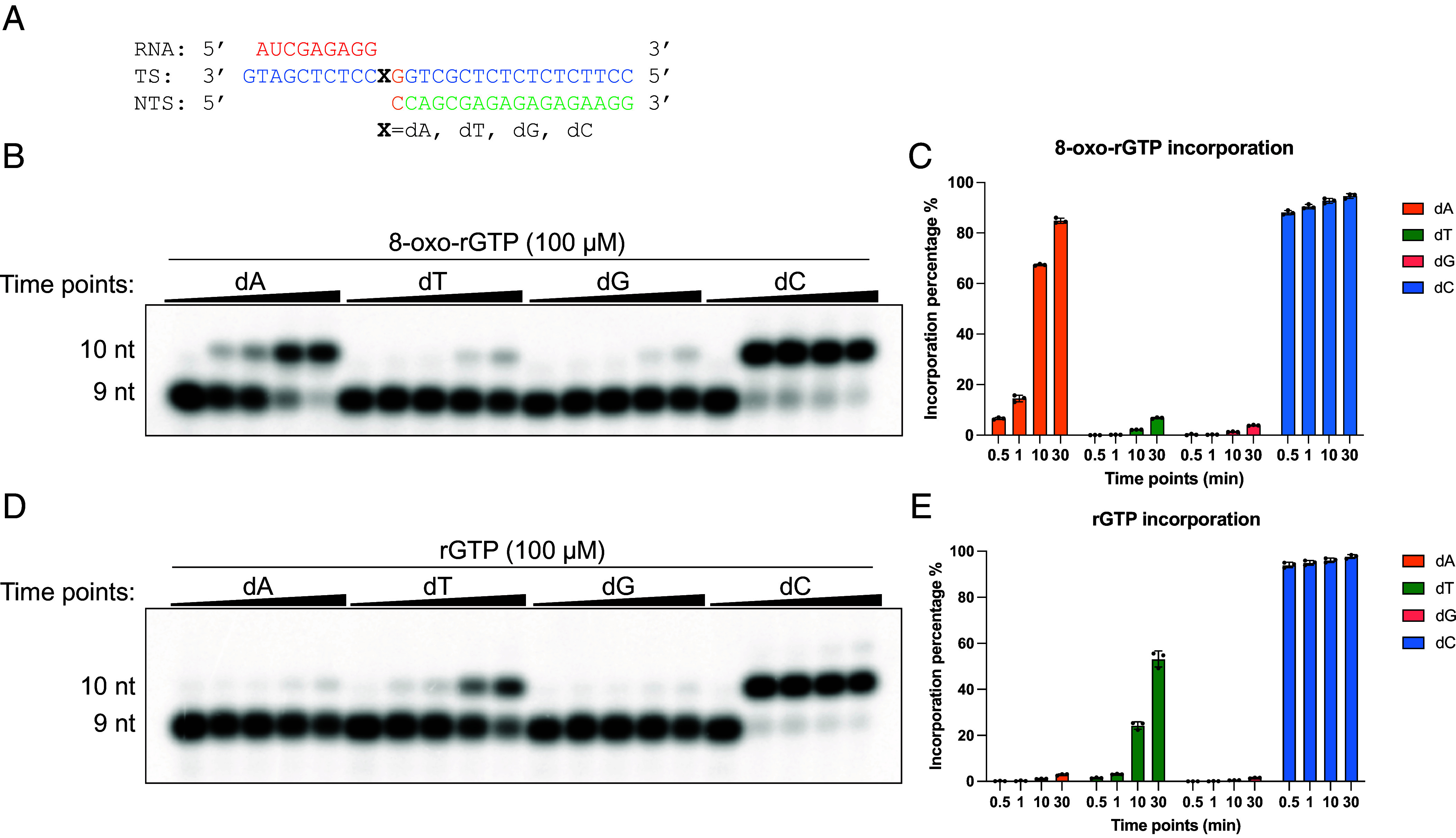
Pol II 8-oxo-rGTP incorporation efficiency and kinetics opposite of four different deoxyribonucleosides. (*A*) Scheme of miniscaffold substrates in 8-oxo-rGTP incorporation assay. (*B*) Incorporation efficiency of 8-oxo-rGTP (100 μM) into the RNA strand opposite dA, dT, dG, or dC in the DNA template. Time points for transcription assays are 0 min, 0.5 min, 1 min, 10 min, and 30 min. (*C*) Quantitative analysis of 8-oxo-rGTP incorporation efficiency at 100 μM concentration. (*D*) Incorporation efficiency of rGTP (100 μM) into the RNA strand opposite four different deoxyribonucleosides as control. Time points for transcription assays are 0 min, 0.5 min, 1 min, 10 min, and 30 min. (*E*) Quantitative analysis of rGTP incorporation efficiency at 100 μM concentration. Note: Quantitative analysis is the average ± SD of three repeats.

To understand the impact of 8-oxo-rGTP incorporation on Pol II transcription, we assembled Pol II elongation complex (EC) with synthetic DNA scaffolds in vitro to examine the selectivity and the incorporation efficiency of 8-oxo-rGTP opposite four natural templates (Fig. 2 *A*–*E*). We observed that Pol II can incorporate 8-oxo-rGTP opposite both dA and dC templates, with a substantially higher efficiency at templates containing dC than dA (Fig. 2 *B* and *C*). This is consistent with previous findings that 8-oxoG is capable of forming a Watson–Crick base pair with C and a Hoogsteen pair with A. In contrast, the selectivity of rGTP is much stricter: It is efficiently incorporated only when the template base is dC. A small amount of rGTP incorporation is detected at dT, due to the formation of a G:T mismatch.

To quantitatively measure the incorporation efficiency of 8-oxo-rGTP opposite dA or dC, we performed presteady state single-turnover transcription assays and determined the kinetic parameters k_pol_ (catalytic rate constant) and K_d, app_ (apparent dissociation constant) for 8-oxo-rGTP:dA, 8-oxo-rGTP:dC, rGTP:dA, and rGTP:dC incorporation (Table 1). Intriguingly, the incorporation efficiency (measured by k_pol_/ K_d, app_) of 8-oxo-rGTP at dC is comparable to that of undamaged rGTP at dC, indicating that Pol II readily incorporates the oxidized nucleotide when paired with cytosine.

**Table 1.** Kinetic parameters of 8-oxo-rGTP:dA, 8-oxo-rGTP:dC, rGTP:dA, and rGTP:dC incorporation

Template	k_pol_ (min^−1^)	K_d, app_ (μM)	k_pol_/K_d, app_ (μM^−1^min^−1^)	Relative efficiency	Template discrimination (relative to 8-oxo-rGTP:dA)	Template discrimination (relative to rGTP:dA)
8-oxo-rGTP:dA	5.8 ± 0.3	2100 ± 300	(2.8 ± 0.4) × 10^−^^3^	1	1	-
8-oxo-rGTP:dC	34 ± 2	5.1 ± 1.5	6.7 ± 2.0	(2.4 ± 0.8) × 10^3^	(2.4 ± 0.8) × 10^3^	-
rGTP:dA	0.019 ± 0.002	1000 ± 300	(1.9 ± 0.6) × 10^−5^	(6.8 ± 2.4) × 10^−3^	-	1
rGTP:dC^*^	980 ± 80	300 ± 37	3.3 ± 0.5	(1.2 ± 0.3) × 10^3^	-	(1.7 ± 0.6) × 10^5^

Note: K_d, app_ is the apparent dissociation constant; k_pol_ is the polymerization rate constant; incorporation efficiency is defined as k_pol_/K_d, app_; relative efficiency is normalized to the reference substrate.

^*^The kinetic parameters of rGTP:dC are from previous literature (27).

Comparing misincorporation events of 8-oxo-rGTP vs. GTP opposite the dA template, we found that 8-oxo-rGTP incorporation efficiency (k_pol_/ K_d, app_) is ~150-fold more efficient than rGTP. The K_d, app_ values of the two substrates at dA differ only modestly (~twofold), whereas the k_pol_ for 8-oxo-rGTP is ~300-fold higher than for rGTP, indicating that the enhanced incorporation reflects a substantially faster chemistry step rather than improved binding.

To understand the template discrimination power for a given substrate, we calculate the ratio of incorporation efficiency of substrate opposite correct template [for example, GTP incorporation efficiency for dC template, (k_pol_/ K_d, app_)_dC_] vs. that for incorrect template [dA template (k_pol_/ K_d, app_)_dA_].

To assess the template discrimination power for a given substrate, we calculated the ratio of its incorporation efficiency opposite the correct template [e.g., GTP incorporation opposite dC, (k_pol_/ K_d, app_)_dC_] to that opposite the incorrect template [dA, (k_pol_/ K_d, app_)_dA_]. For GTP, dC/dA template discrimination power is ~1.7 × 10^5^. In contrast, for 8-oxo-rGTP, the dC/dA template discrimination power is reduced to ~2,400, corresponding to a reduction of over 70-fold.

Taken together, these data demonstrate that transcription-coupled RNA damage by incorporation of 8-oxo-rGTP can contribute a significant level for RNA damage (at least proportional to the ratio of 8-oxo-rGTP/rGTP in the nucleotide pool). Pol II efficiently incorporates 8-oxo-rGTP at dC with kinetics similar to those of undamaged rGTP incorporation. In addition, oxidation of the GTP pool also greatly increases error-prone incorporation opposite a dA template.

### Extension Step: Pol II Extends More Efficiently from an 8-Oxo-rG:dC Pair Over an 8-oxo-rG:dA Pair.

**Fig. 3.**
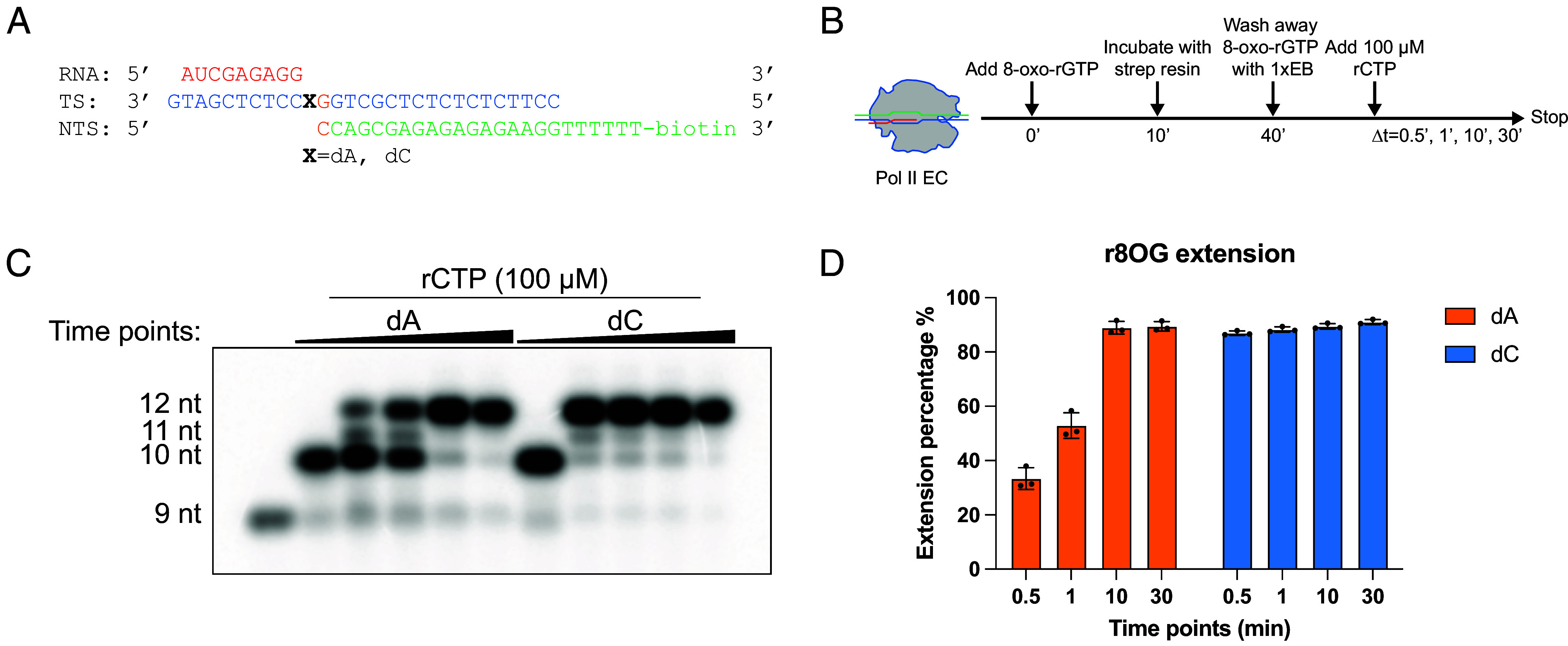
The effect on Pol II transcription elongation after r8OG incorporation into RNA. (*A*) The scheme of miniscaffolds substrates in transcription assays. (*B*) Experimental set up to investigate the effect on Pol II transcription elongation when r8OG is positioned at the 3′ end of the nascent RNA chain. The RNA strand bearing a terminal r8OG is generated through in vitro transcription by adding r8OG into Pol II EC and reacting for 10 min. (*C*) Transcription elongation efficiency of Pol II ECs containing a newly incorporated r8OG in the RNA strand paired with dA or dC. Time points for transcription assays are 0 min, 0.5 min, 1 min, 10 min, and 30 min. (*D*) Quantitative analysis of rCTP incorporation efficiency (100 μM) immediately following r8OG incorporation into RNA strand opposite dA or dC. Note: Quantitative analysis is the average ± SD of three repeats.

To investigate how newly incorporated 8-oxo-rG at the 3′-end of RNA affects downstream Pol II elongation, we established an in vitro extension assay (Fig. 3 *A* and *B*) using miniscaffolds containing either dA or dC at the +1 template position. To introduce a single 8-oxo-rG (r8OG) at the 3′-end of the RNA, we used scaffolds in which the nontemplate DNA strand was 3′-biotinylated. Pol II ECs were assembled and incubated with 8-oxo-rGTP to allow incorporation at the RNA terminus. The resulting complexes were then captured on streptavidin magnetic beads, followed by extensive washing with an elongation buffer to remove unincorporated 8-oxo-rGTP, ensuring that subsequent extension was not influenced by free 8-oxo-rGTP nucleotides.

Pol II transcript extension was initiated by adding 100 μM rCTP (matched with the next template base). When the terminal r8OG was paired with dC, Pol II extends efficiently: ~90% of the complexes incorporated rCTP within 30 s. In contrast, a terminal r8OG:dA mismatch markedly slowed extension, with only ~30% rCTP incorporation after 30 s. These observations indicate that r8OG at the 3′-end of RNA selectively impedes Pol II elongation when it is mispaired with dA, whereas the r8OG:dC pair supports efficient transcription extension.

### Proofreading Step: r8OG:dC Is More Susceptible to TFIIS-Stimulated RNA Cleavage than R8OG:dA.

Nucleotide misincorporation leads to Pol II pausing and backtracking. The transcription factor TFIIS plays a critical role in proofreading by stimulating the intrinsic endonucleolytic cleavage activity of Pol II that removes misincorporated nucleotides. Through this proofreading mechanism, TFIIS enhances transcription fidelity and facilitates recovery from transcriptional arrest, particularly in the presence of DNA lesions or oxidative damage.

**Fig. 4.**
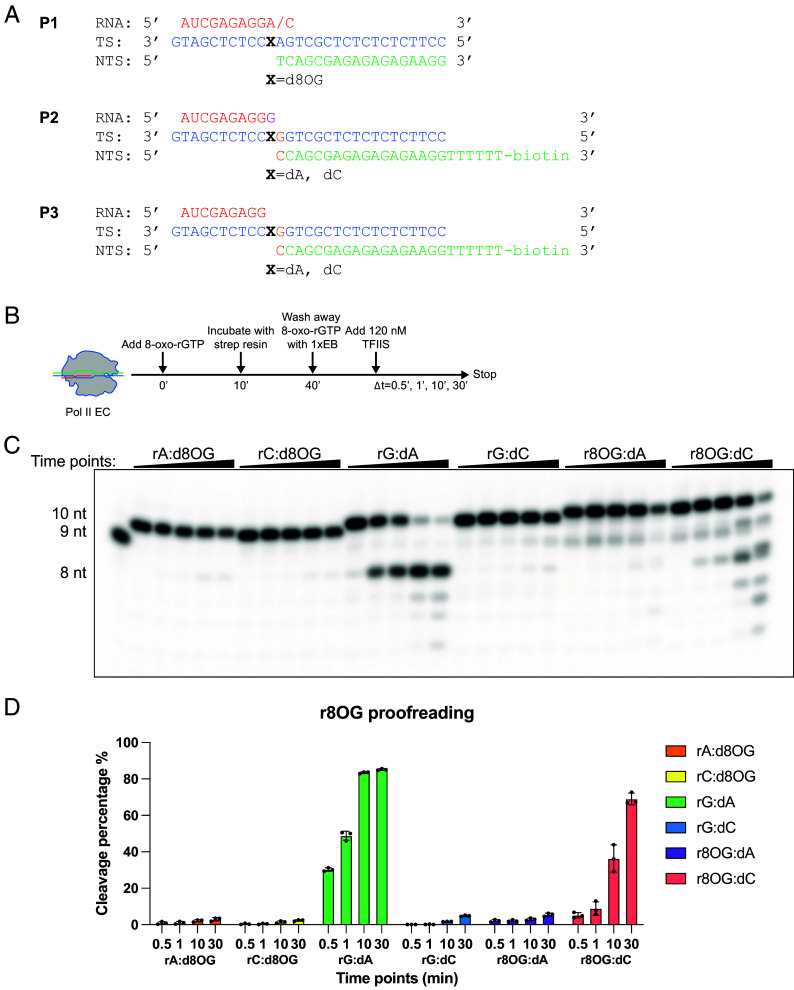
Efficiency of TFIIS-stimulated Pol II proofreading-mediated cleavage of newly incorporated r8OG in the RNA strand. (*A*) Schematic representation of miniscaffold substrates used in the proofreading assay. Miniscaffolds P1 and P2 are control substrates for P3. P1 contains a d8OG at +1 position on the template strand as a control of P3 which bears a r8OG at +1 position on the RNA strand. P2 contains a natural G at +1 site on the RNA strand, serving as the G vs. r8OG control for P3, where r8OG occupies the same RNA position. (*B*) Experimental design for investigating TFIIS-stimulated Pol II proofreading cleavage on miniscaffold P3. For miniscaffold P1, the first two steps, addition of 8-oxo-rGTP and incubation with strep resin are omitted. For TFIIS stimulated Pol II proofreading assay on P2, the addition of 8-oxo-rGTP is not performed. (*C*) Page gel analysis of TFIIS-stimulated Pol II proofreading on miniscaffolds with different base-pairing configurations. The first two panels correspond to assays performed on miniscaffold P1, the third and fourth panels on miniscaffold P2, the last two panels on miniscaffold P3. Time points for proofreading assays are 0 min, 0.5 min, 1 min, 10 min, and 30 min. (*D*) Quantitative analysis of TFIIS-stimulated proofreading cleavage efficiency on different base pairings. Note: Quantitative analysis is the average ± SD of three repeats.

To examine whether 8-oxo-rGTP incorporation into RNA transcript triggers TFIIS-stimulated cleavage, we established an in vitro proofreading assay (Fig. 4 *A* and *B*). Two sets of controls were included: 1) rA:d8OG and rC:d8OG, in which 8-oxoG resides on the template strand, serving as controls for template lesions; 2) rG:dA and rG:dC, in which undamaged rG is positioned at the RNA 3′-terminus, serving as controls for unmodified RNA ends. For scaffolds containing r8OG at the RNA 3′-end, Pol II ECs were preincubated with 100 μM 8-oxo-rGTP for 10 min to ensure r8OG incorporation at the RNA 3′-end. Other scaffolds were assembled directly using presynthesized 10-nt RNAs. TFIIS was then added to 120 nM to initiate cleavage.

As previously reported, Pol II EC with rA:d8OG and rC:d8OG showed no detectable cleavage, consistent with their ability to escape Pol II proofreading. For the case of undamaged rG, no cleavage occurred when paired with dC; however, the rG:dA mismatch generated an 8-nt cleavage product (n-2). This signature n-2 product indicates that the mismatched rG undergoes 1-nt backtracking and extrudes into the secondary channel, where it is subsequently cleaved in a TFIIS-dependent manner (28).

Remarkably, when 8-oxoG is located at the RNA 3′-end, the cleavage patterns differ substantially from those observed in Pol II ECs with template lesion-containing scaffolds or nondamaged scaffolds. By comparing with rG:dC pair or rC:d8OG pair, the r8OG:dC pair is more susceptible to TFIIS-stimulated cleavage, especially with prolonged incubation (~70% cleavage after 30 min), producing multiple shortened RNA products. In contrast, the r8OG:dA pair exhibits minimal cleavage (<10%) and primarily produces a 9-nt product, suggesting a minor cleavage event without prior backtracking. This is in sharp contrast with the robust n-2 cleavage product observed in the rG:dA pair. Taken together, these results indicate that the presence of r8OG at the RNA 3′-end also greatly interferes with Pol II proofreading activity. r8OG:dA pair is more resistant to TFIIS-stimulated cleavage than that for the r8OG:dC pair. As a result, more error-prone r8OG-containing transcripts escape the surveillance of TFIIS-mediated cleavage.

### 8-oxo-rGTP Occupies the A-Site Prior to Incorporation and Adopts an Anti-conformation When Pairing with dC.

To investigate how Pol II recognizes 8-oxo-rGTP as a substrate and whether 8-oxo-rGTP exhibits different behaviors during incorporation opposite dA and dC, we solved four crystal structures of Pol II containing a dA or a dC at +1 site in the template strand DNA soaked with 8-oxo-rGTP using the miniscaffold shown in Fig. 2*A* (29–32).

**Fig. 5.**
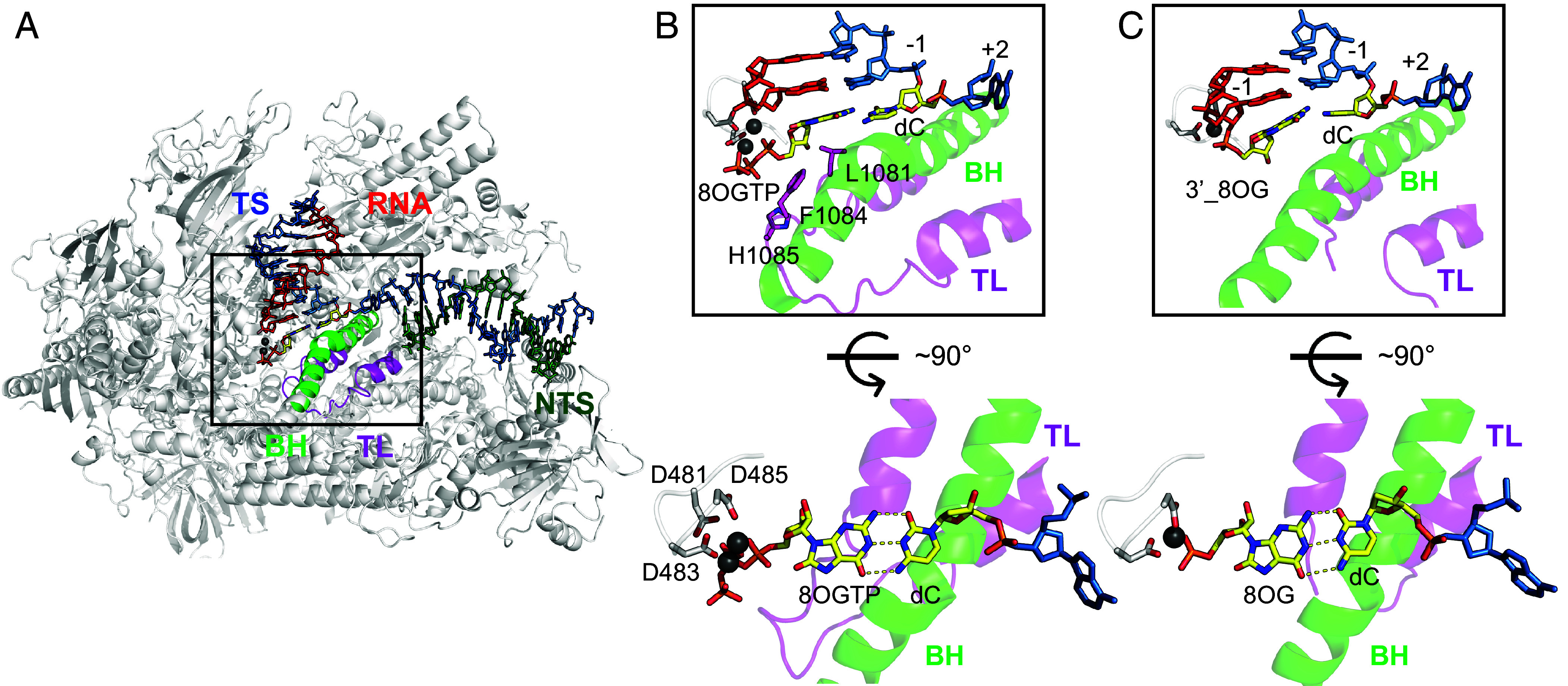
Interactions of 8-oxo-rG with dC before and after incorporation. (*A*) Overall structure of Pol II EC with dC at +1 position and 8-oxo-rGTP (r8OGTP) opposite dC. Portions of Rpb1 and Rpb2 are omitted to show the catalytic center. (*B*) Catalytic center of Pol II with r8OGTP bound at the addition site (A site) and paired with dC at +1 position before reaction. r8OGTP adopts the anti-conformation when paired with dC and forms a canonical Watson–Crick base pair. The Pol II TL is fully closed, stabilizing 8OGTP at the A site. (*C*) Catalytic center of Pol II with r8OGTP incorporated and paired with dC at +1 position after reaction. The newly incorporated r8OG forms a Watson–Crick base pair with dC at +1 position.

First, to capture the substrate binding state (prechemistry state) of the 8-oxo-rGTP opposite dC template, we used 3′deoxy RNA primer to prevent incorporation reaction. We found that 8-oxo-rGTP is bound at the canonical addition site (A-site) and forms a Watson–Crick base pair with dC in anti-conformation (Fig. 5 *A* and *B*), which can also be observed in other 8OG:C base pair containing structures (6, 7, 33–36). Meanwhile, there is extra electron density in the active site, which is attributed to the fully closed TL that interacts with 8-oxo-rGTP. Pol II residues L1081, F1084, and H1085 on the TL help to stabilize 8-oxo-rGTP at the active site while paired with dC except for the hydrogen bound formed between two nucleobases. Same interactions are also observed in Pol II EC with cognate undamaged nucleotide in the active site (37).

To investigate the postchemistry state immediately after 8-oxo-rGTP incorporation, we soaked Pol II EC containing regular RNA primer with 5 mM 8-oxo-rGTP with the same concentration of Mg^2+^ to allow nucleotide incorporation to proceed to completion in the crystal. Pol II EC structure is captured after 8-oxo-rGTP being incorporated and stayed at the pretranslocation state (Fig. 5*C*). We found that the newly incorporated r8OG forms a Watson–Crick base pair with dC in anti-conformation. Taken together, 8-oxo-rGTP, similar to undamaged GTP, preferentially occupies the A-site prior to incorporation, forms Watson–Crick base pair with dC, and promotes the closure of the TL. This in turn facilitates the chemistry reaction to incorporate 8-oxo-rGTP into the RNA chain. The structures of Pol ECs contain 8-oxo-rG:dC (pre- and postchemistry state) offer a mechanistic explanation for the kinetics observation that 8-oxo-rGTP is incorporated with an efficiency similar to that of undamaged GTP.

### 8-Oxo-rGTP Occupies the E-Site Prior to Incorporation and Adopts a Syn-Conformation When Pairing with dA.

To investigate how Pol II recognizes and incorporates 8-oxo-rGTP opposite dA template, we also determined Pol II EC structures containing 8-oxo-rGTP opposite dA template in both the substrate binding state (prechemistry) and incorporation state (postchemistry) in a similar manner.

**Fig. 6.**
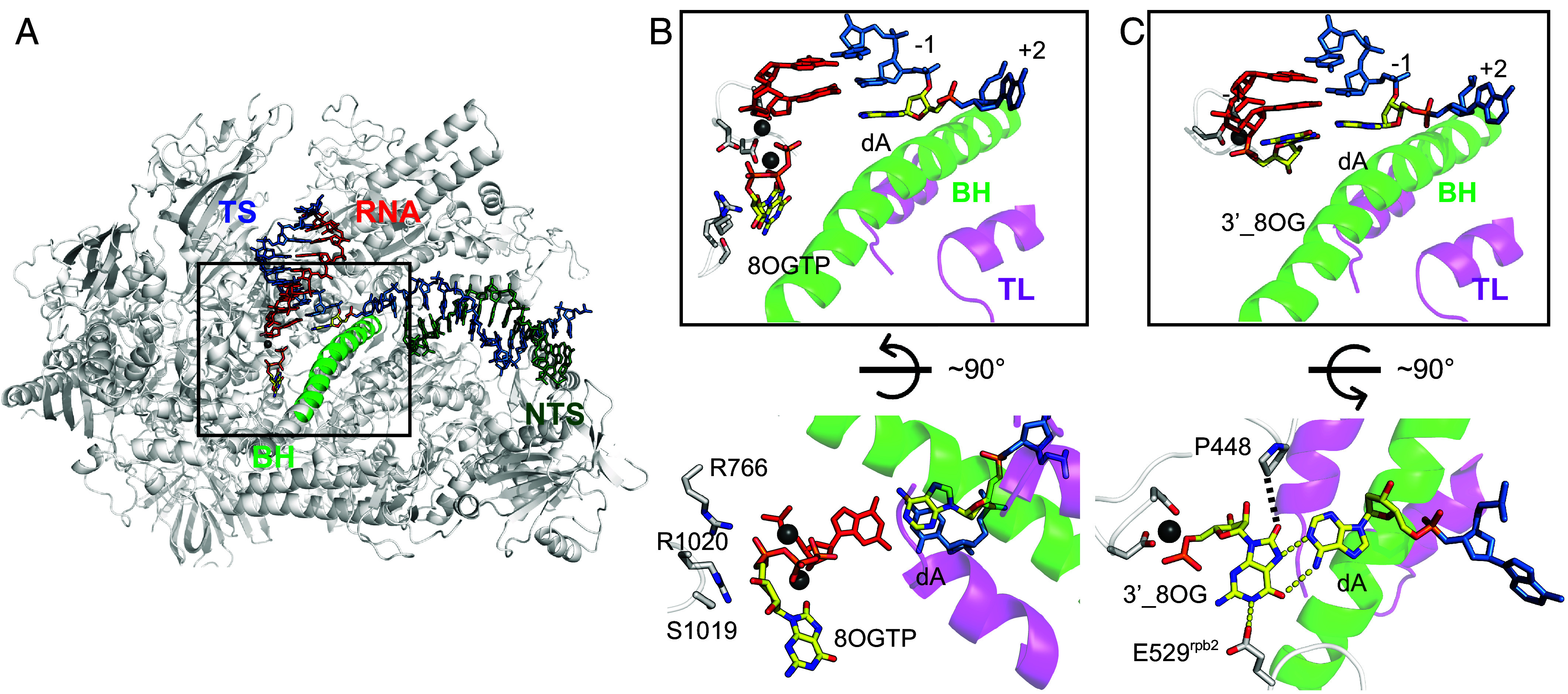
Interactions of 8-oxo-rG with dA before and after incorporation. (*A*) Overall structure of Pol II EC with dA at +1 position and 8-oxo-rGTP at the entry site (E site). Portions of Rpb1 and Rpb2 are omitted to show the catalytic center. (*B*) Catalytic center of Pol II with 8-oxo-rGTP bound at the E site before reaction. (*C*) Catalytic center of Pol II with 8-oxo-rGTP incorporated into RNA strand and paired with dA at +1 position after chemistry reaction. The newly incorporated r8OG adopts the syn-conformation and forms a Hoogsteen base pair with dA at +1 position. Yellow dashed lines indicate hydrogen bonds, whereas the black dashed line indicates a potential Van der Waals interaction.

For the substrate binding state (prechemistry), we found that the 8-oxo-rGTP substrate binding site differs strikingly between Pol II EC:dA complex and Pol II EC:dC complex, placing its nucleobase too far from the templating dA (Fig. 6 *A* and *B*). The TL is in an open state. This result is consistent with relative slow K_pol_ and weak binding affinity of 8-oxo-rGTP (high K_d, app_ value).

To understand how 8-oxo-rG pair with dA template after chemistry reaction, we determined the structure of postchemistry state allowing 8-oxo-rGTP to be incorporated into Pol II EC:dA complex crystal. We found that Pol II EC is captured in the pretranslocation state and the newly incorporated 8-oxo-rG forms Hoogsteen base pair with dA at +1 site in a syn-conformation (Fig. 6*C*). This result indicates that there is an equilibrium from E-site to A-site that allows 8-oxo-rGTP to reach to A-site for nucleotide incorporation. The structures of Pol II EC containing 8-oxo-rGTP:dA (pre- and postchemistry states) provide the structural explanation to why 8-oxo-rGTP incorporation opposite dA is much slower than opposite dC template.

**Fig. 7.**
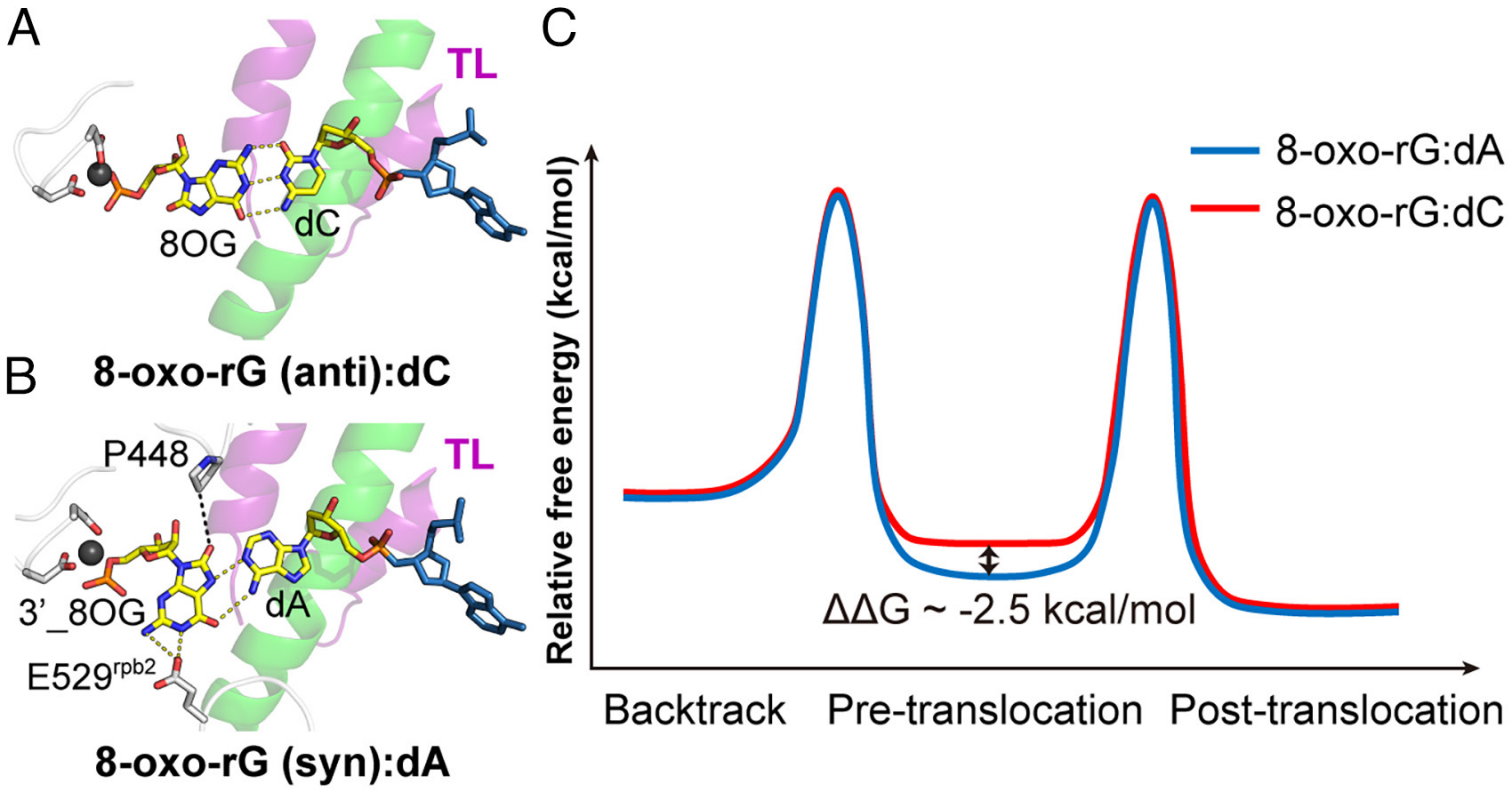
E529-mediated translocation control of Pol II after 8-oxo-rG:dA formation. (*A*) Postchemistry active site for 8-oxo-rG:dC. (*B*) Postchemistry active site for 8-oxo-rG:dA showing the E529 contact. (*C*) Schematic energy landscape of Pol II translocation. The 8-oxo-rG:dA complex has a lower energy state created by E529 and RNA stacking. The stabilization energy (ΔΔG ~ −2.5 kcal/mol) denotes the calculated energy difference between the syn- and anti-conformation (*SI Appendix*, Table S7 and *Materials and Methods*).

The postchemistry structure places the newly added 8-oxo-rG in syn-conformation and pairs it with template dA in a Hoogsteen geometry at +1 position. Interestingly, we found that the syn-8-oxo-rG is positioned to hydrogen bond with Rpb2 E529 in fork loop 2 and the complex remains in a pretranslocation register (Fig. 6*C*). This specific syn-8-oxo-rG:E529 contact may stabilize a pretranslocation configuration that is unfavorable for motion in either forward or reversed direction (*SI Appendix*, Fig. S4). To quantify the stability of this unique conformation, we performed structure-based energy calculations (Fig. 7 and *Materials and Methods*). The analysis revealed that the syn-conformation is thermodynamically stabilized by approximately ~2.5 kcal/mol relative to the anticonformation within the active site (*SI Appendix*, Table S7). This net stabilization arises from three distinct interactions. Two interactions favor the syn-conformation: enhanced base stacking with the RNA 3′-end (ΔΔG ≈ −1.61 kcal/mol) and a specific side-chain interaction with Rpb2 (ΔΔG ≈ −1.50 kcal/mol). In contrast, the interaction with the template DNA slightly favors the anti-conformation (ΔΔG ≈ +0.63 kcal/mol), which is consistent with the ability of 8-oxoG in the anti-conformation to form more favorable Watson–Crick-like base pairing and hydrogen-bonding interactions. Taken together, these results explain why the 8-oxo-rG:dA pair is resistant to backtracking and TFIIS-stimulated cleavage and exhibits slow extension in biochemical assays.

## Discussion

Oxidative stress causes oxidative damage in both nucleic acids and nucleotide pool. In this study, we elucidated how oxidized nucleotide pool interferes with eukaryotic transcription by Pol II and contributes RNA damage in a transcription-coupled manner. Using single-turnover nucleotide incorporation assay, we quantitatively compared Pol II incorporation of 8-oxo-rGTP with that of undamaged rGTP on both dC and dA templates. Remarkably, incorporation of 8-oxo-rGTP opposite a dC template occurs with an efficiency comparable to that of nondamaged GTP, indicating that when dC is present at the template position, oxidized and nonoxidized guanine nucleotides have a similar probability of being incorporated into RNA.

Notably, the cellular level of 8-oxo-rGTP has been estimated to be ~0.02% of total GTP under normal conditions and can increase to approximately 2 to 5% under modest oxidative stress (21, 22). Considering the near-equivalent incorporation efficiencies of 8-oxo-rGTP and rGTP on dC templates, these measurements suggest that substantial RNA damage can be introduced in transcription-coupled manner by incorporation of oxidized guanine nucleotides from the nucleotide pool.

In contrast, although incorporation of 8-oxo-rGTP opposite dA is approximately three orders of magnitude less efficient than 8-oxo-rGTP incorporation opposite dC, it is enhanced by ~150-fold compared with the mismatched incorporation of rGTP opposite dA, substantially increasing the likelihood of oxidized nucleotide misincorporation relative to canonical mismatch events. Importantly, Pol II proofreading does not effectively counteract incorporation of 8-oxo-rGTP. Both 8-oxo-rG:dC and 8-oxo-rG:dA pairs exhibit low cleavage efficiencies, with the 8-oxo-rG:dA pair being unexpectedly more resistant to TFIIS-stimulated cleavage. Moreover, extension assays demonstrate that Pol II can efficiently resume elongation following 8-oxo-rGTP incorporation, with particularly high extension efficiency from 8-oxo-rG:dC pairs and appreciable extension from 8-oxo-rG:dA pairs. Together, these results indicate that Pol II can tolerate 8-oxo-rGTP incorporation on both dC and dA templates through distinct kinetic trade-offs: efficient incorporation and extension in the case of dC and reduced proofreading surveillance in the case of dA. As a consequence, oxidized 8-oxo-rGTP can be embedded into RNA transcripts across multiple template contexts.

Transcription-coupled RNA damage by incorporation of 8-oxo-rGTP creates a systematic threat to the transcriptome, which is distinct from genomic DNA lesions, as it bypasses canonical DNA damage surveillance and directly propagates oxidative damage into functional RNA molecules (38, 39). The resulting accumulation of 8-oxoguanine within RNA transcript would compromise the integrity and function of both noncoding RNAs and messenger RNAs, with downstream consequences for RNA processing, translational fidelity, and proteostasis (38).

Our structural studies revealed distinct modes of recognition and incorporation of 8-oxo-rGTP opposite dC and dA templates. On a dC template, 8-oxo-rGTP preferentially occupies the A-site, where it forms a canonical Watson–Crick base pair with dC and promotes TL closure. In this context, the oxidized nucleotide remains in the anti-conformation before and after catalysis, closely mimicking the geometry of correct rGTP incorporation and rationalizing the near-equivalent incorporation and extension efficiencies observed in our kinetic assays. In contrast, when the template base is dA, 8-oxo-rGTP prefers to be at the E-site in an anti-conformation before chemistry reaction, and we do not capture an A-site–bound complex in which 8-oxo-rGTP forms a Hoogsteen base pair with dA prior to chemistry. This structural observation is fully consistent with our kinetic measurements, which show that the apparent binding affinity (K_d, app_) of 8-oxo-rGTP opposite dA is substantially weaker than that opposite dC. Notably, once incorporated into the RNA chain, the r8-oxo-GMP adopts a syn-conformation and forms a Hoogsteen base pair with the dA template. This indicates that incorporation of 8-oxo-rGTP opposite dA is accompanied by a pronounced conformational rearrangement of the oxidized nucleotide, resulting in stabilization of the Hoogsteen geometry in the postchemistry state.

This behavior closely parallels previous observations in which 8-oxoG resides in the DNA template strand, where rATP preferentially occupies the E-site rather than forming a stable Hoogsteen pair with template 8-oxoG at the A-site. In that context, the template 8-oxoG undergoes interconversion between anti- and syn- conformations, and only the syn-conformation state supports Hoogsteen pairing with rATP and subsequent incorporation (6). Together, these findings highlight a shared mechanistic principle in which the intrinsic conformational plasticity of 8-oxoguanine, whether in the incoming ribonucleotide or in the template strand, governs productive misincorporation by Pol II. However, despite these structural similarities, a critical distinction emerges at the Pol II proofreading checkpoint: For the 8-oxo-dG DNA lesion, the resulting pair of rC:d8OG and rA:d8OG can completely escape Pol II proofreading (Fig. 4 *C* and *D*) (40). On the other hand, the resulting r8OG:dC pair incorporated from 8-oxo-rGTP is susceptible to slow TFIIS-stimulated Pol II cleavage (~70% at 30 min, Fig. 4 *C* and *D*), whereas the r8OG:dA pair remains quite resistant (which is likely due to stabilization of r8OG:dA by E529). This difference likely arises from the asymmetric environment experienced by template 8-oxoG vs. incoming 8-oxo-rG within the Pol II catalytic center.

Our results also begin to place Pol II into a broader framework of substrate-level oxidation across polymerase families. Structural studies of DNA polymerase μ demonstrated that 8-oxo-rGTP can be efficiently incorporated during double-strand break repair and captured a ternary complex in which 8-oxo-rGTP adopts a syn-conformation and forms a Hoogsteen pair with a template adenine (25). That work established that oxidized ribonucleotides can serve as proficient substrates for polymerases and that Hoogsteen base pairing represents a shared structural solution for accommodating 8-oxoguanine lesions within the active site. Notably, while substrate-level oxidation can be accommodated through similar base-pairing geometries in different polymerases, the functional consequences are shaped by the local architecture and biological context of the active site. In the case of a repair polymerase such as Pol μ, this tolerance facilitates lesion bypass during DNA repair, whereas in RNA Pol II, analogous structural accommodation enables oxidative damage to be directly embedded into RNA transcript, posing a distinct threat to transcription fidelity, thereby compromising downstream RNA processing, translation fidelity, and the integrity and function of noncoding RNAs.

Previous work identified E529 residue as a regulator of elongation and translocation (41). Mutational analyses of fork loop 2 have shown that E529 and neighboring residues modulate the rate and translocation behavior of Pol II on undamaged templates. Our structures now reveal how an oxidized ribonucleotide can directly interact with E529 and a novel role of E529 in regulating Pol II translocation via its specific interactions with *syn*-8oxo-rG. The hydrogen bond network between 8-oxo-rG and E529 stabilizes a specific geometry that favors the pretranslocation state and disfavors translocation toward either the posttranslocation or backtracked state (Fig. 7). This provides a direct structural explanation to how a single amino acid side chain can act as a regulator during the translocation. It also suggests that fork loop 2 is not only a passive sensor of base pairing at +1 but can become an active participant in lesion-specific control of Pol II elongation dynamics. E529 variants that weaken these hydrogen bonds would be expected to reduce pausing by 8-oxo-rG opposite dA, while variants that strengthen or mimic this interaction might sensitize the enzyme to other noncanonical base pairs at +1.

Emerging evidence showed significant elevated levels of 8-oxo-rG are presented in RNA transcripts upon oxidative stress or cell aging (26, 42–44). While these RNA lesions can be introduced posttranscriptionally (45–47), our studies suggest that incorporation of oxidized nucleotide from nucleotide pool could be an important source for transcription-coupled RNA damage. Unlike 8-oxo-dGTP, which is efficiently sanitized from the nucleotide pool by MTH1, 8-oxo-rGTP is a relatively poor substrate for MTH1-mediated hydrolysis in mammalian cells (48, 49). As a result, oxidized ribonucleotides such as 8-oxo-rGTP are more likely to persist in the cellular NTP pool in mammalian cells and encounter RNA polymerase II during transcription. This limited preincorporation sanitation places a greater burden on transcriptional fidelity and proofreading mechanisms to cope with oxidized ribonucleotide incorporation.

Accumulating evidence indicates that oxidation of the ribonucleotide pool represents an underappreciated upstream source of RNA damage with broad biological consequences. Under oxidative stress, guanine nucleotides can be oxidized to 8-oxo-rGTP and incorporated into nascent RNA transcripts by RNA polymerases. Once embedded in mRNA, 8-oxo-rGTP perturbs base pairing–dependent processes such as pre-mRNA splicing and the function of regulatory noncoding RNAs. At the translational level, oxidized RNAs promote ribosome stalling, leading to miscoding and translational stress (38). Consistent with these molecular defects, it is reported that accumulation of 8-oxoG–containing mRNAs in MTH1-deficient mammalian cells has been linked to proteotoxic outcomes, including enhanced aggregation of amyloidogenic peptides, suggesting a potential mechanistic connection between oxidative RNA damage and disease-associated protein misfolding (26). Together with our findings that Pol II can efficiently incorporate 8-oxo-rGTP from the nucleotide pool during transcription, these observations establish a mechanistic link between oxidative stress and transcription-coupled RNA damage, which in turn leads to defective translation and disease-associated cellular dysfunction, particularly in the contexts of aging and neurodegeneration.

## Materials and Methods

### Purification of *S. Cerevisiae* Pol II.

Saccharomyces cerevisiae Pol II comprising 10 subunits without Rpb4/7 and carrying a protein-A tag at the Rpb3 subunit (strain kindly provided by the Roger Kornberg laboratory) was purified as described. Briefly, ~40 L of harvested yeast cells were lysed using a microfluidizer. Cell debris was removed by centrifugation, and nucleic acids were precipitated with polyethylenimine. The clear supernatant was subjected to ammonium sulfate precipitation, and the resulting pellet containing Pol II was resuspended and incubated with IgG resin. After tobacco etch virus protease cleavage, Pol II was eluted from the IgG resin (GE Healthcare) and further purified using Heparin and Q ion-exchange columns (GE Healthcare). The major peak containing Pol II was collected, concentrated to ~5 μM, and stored at –80 °C.

### Transcription Incorporation and Extension Assay with *S. Cerevisiae* Pol II.

The RNA primers and DNA oligonucleotides in this study were purchased from IDT, while the DNA oligonucleotide containing a d8OG modification was obtained from TriLink. The detailed sequences of all RNA primers and DNA oligonucleotides are provided in Figs. 2*A*, 3*A*, and 4*A*, and the *SI Appendix*.

Scaffolds for all assays were assembled by annealing 200 nM 5′-^32^P-labeled RNA primer, 400 nM template DNA strand, and 600 nM nontemplate DNA strand in 1× Elongation Buffer (20 mM Tris-HCl, pH 7.5, 40 mM KCl, 5 mM MgCl_2_ and 5 mM DTT).

For the incorporation assay, 240 nM 10-subunit Pol II was preincubated with 40 nM scaffold at room temperature for 10 min in 1× Elongation Buffer to form the Pol II EC. The EC was then mixed with an equal volume of 200 μM 8-oxo-rGTP or rGTP in 1× EB lacking 5 mM DTT to initiate the reaction. At the indicated time points, aliquots of the reaction mixture were withdrawn and quenched with a quenching buffer (90% formamide, 50 mM EDTA, 0.05% xylene cyanol, and 0.05% bromophenol blue).

For the extension assay, 8-oxo-rGTP was first incorporated into the 3′ end of the RNA strand using scaffolds assembled with a 3′-biotin-labeled nontemplate DNA strand. After Pol II EC assembly, the reaction was started by adding an equal volume of 200 μM 8-oxo-rGTP and incubating for 10 min to incorporate 8-oxo-rG at the RNA 3′ end. The reaction mixture was then incubated with Strep resin at room temperature for 30 min, then washed 3× with 1× EB to remove unincorporated 8-oxo-rGTP, and resuspended in 1× EB. The resin suspension was mixed with an equal volume of 200 μM rCTP in 1× EB lacking 5 mM DTT to initiate the transcription reaction. At the indicated time points, aliquots were quenched as described above.

All samples were denatured at 95 °C for 10 min, resolved on 12% denaturing urea gels, and visualized using an Amersham Typhoon Biomolecular Imager (GE Healthcare). Substrate and product bands were quantified with Image Lab (Bio-Rad) and analyzed with GraphPad Prism.

### TFIIS-Stimulated Proofreading Cleavage Assay.

The detailed scaffold information is provided in Fig. 4*A* and the *SI Appendix*. Scaffolds were assembled as described above. For the TFIIS-stimulated cleavage assay, three types of scaffolds were used (P1, P2, and P3; Fig. 4*A*), which differ slightly in design.

For the P1 scaffold, 10-subunit Pol II was preincubated with P1 at room temperature for 10 min to assemble the Pol II EC, as described above. The proofreading cleavage reaction was initiated by adding an equal volume of 240 nM TFIIS diluted in 1× EB.

For the P2 scaffold, which contains a 3′-biotin-labeled nontemplate DNA strand, the Pol II EC was assembled in the same way and incubated with Strep resin for 30 min. The resin was then washed three times with 1× EB to remove unbound Pol II EC. The proofreading reaction was initiated by adding an equal volume of 240 nM TFIIS to the resin-bound Pol II EC.

For the P3 scaffold, after Pol II EC assembly, 8-oxo-rGTP was added to a final concentration of 100 μM and incubated for 10 min to incorporate r8OG at the 3′ end of the RNA strand. The reaction mixture was then incubated with Strep resin for 30 min, followed by three washes with 1× EB to remove unincorporated 8-oxo-rGTP and unbound Pol II EC. The proofreading reaction was started by adding an equal volume of 240 nM TFIIS to the resin-bound complex.

At the indicated time points, aliquots were withdrawn and quenched with a quenching buffer. All samples were denatured at 95 °C for 10 min, resolved on 12% denaturing urea gels, and visualized using an Amersham Typhoon Biomolecular Imager (GE Healthcare). Substrate and product bands were quantified with Image Lab (Bio-Rad) and analyzed using GraphPad Prism.

### Single-Turnover Nucleotide Incorporation Assay and Data Fitting.

The kinetic parameters (k_pol_ and K_d, app_) for 8-oxo-rGTP and rGTP incorporation opposite dA or dC were determined using single-turnover nucleotide incorporation assays. A total of 240 nM yeast 10-subunit Pol II was preincubated with 40 nM scaffold in 1× Elongation Buffer at room temperature for 10 min to form Pol II EC, as described above. Reactions were initiated by adding an equal volume of 8-oxo-rGTP or rGTP at increasing concentrations in 1× Elongation Buffer lacking 5 mM DTT. The concentration ranges were as follows:

8-oxo-rGTP: for the dA template, 2 to 10,000 μM (2, 5, 10, 20, 50, 100, 200, 500, 1,000, 2,000, 5,000, 7,500, and 10,000 μM); for the dC template, 0.25 to 80 μM (0.25, 0.5, 1, 2, 5, 10, 20, 30, 50, and 80 μM).

rGTP: for the dA template, 150 to 10,000 μM (150, 300, 600, 900, 1,200, 2,500, 5,000, 7,500, and 10,000 μM).

At the indicated time points, aliquots were withdrawn and quenched with a quenching buffer. All samples were analyzed as described above, with substrate and product bands quantified using Image Lab (Bio-Rad) and further analyzed using GraphPad Prism.

Nonlinear regression fitting was performed with GraphPad Prism. The time dependence of 8-oxo-rGTP or rGTP incorporation at each concentration was fit to a single-exponential equation to obtain the observed rate constant (k_obs_) for product formation. Subsequently, the dependence of k_obs_ on nucleotide concentration was fit to the Michaelis–Menten equation to derive k_pol_ and K_d, app_.

### Crystallization and Structure Determination.

The scaffolds used for crystallization were essentially the same as those shown in Fig. 2*A*, except that both a normal 9-mer RNA and a 3′-deoxy 9-mer RNA were employed. Crystallization and structure determination were carried out as previously described. Briefly, template DNA (dC template: CCTTCTCTCTCTCGCTGGCCCTCTCGATG; dA template: CCTTCTCTCTCTCGCTGGACCTCTCGATG), nontemplate DNA (CCAGCGAGAGA GAGAAGG), and 9-mer RNA (normal: AUCGAGAGG; 3′-deoxy: AUCGAGAGG-3′deoxy) were mixed at a molar ratio of 1:1.25:1.25 and annealed in 1× Elongation Buffer to form scaffolds. The resulting scaffold was incubated with 10-subunit Pol II on ice for 1 h to assemble the Pol II EC. The buffer was then exchanged to crystallization buffer (25 mM Tris-HCL, pH 7.5, 20 mM NaCl, 5 mM DTT, 1 μM Zn(OAc)_2_ and 100 μM EDTA) using ultrafiltration, and the Pol II EC was concentrated to ~6 mg/mL for crystallization.

Crystals were obtained by the hanging-drop vapor-diffusion method at room temperature under conditions containing 390 mM ammonium phosphate (pH 6.0), 5 mM DTT, 5 mM dioxane, and 9 to 13% PEG 6000, with crystallization proceeding for 7 d. Crystals were then soaked overnight at 4 °C in cryosolution (100 mM MES, pH 6.0, 350 mM NaCl, 5 mM DTT, 5 mM dioxane, 16% PEG 6000, and 17% PEG 400) supplemented with 5 mM 8-oxo-rGTP and 5 mM MgCl_2_.

X-ray diffraction data were collected under cryogenic conditions. Datasets for the 9-mer RNA ECs with dC and dA templates were collected at beamline 5.0.1 of the Advanced Light Source, Lawrence Berkeley National Laboratory. Datasets for the 3’-deoxy RNA ECs with dC and dA templates were collected at beamline 12-2 of the Stanford Synchrotron Radiation Lightsource. All raw data were processed and scaled using XDS (50). All structures were solved by molecular replacement using Phaser from the Phenix software suite, with the undamaged 10-subunit Pol II structure (PDB ID: 6UQ2) as the search model (51, 52). All crystals belonged to the space group C2, with one EC in the asymmetric unit. Model building and refinement were performed through iterative cycles of manual model building in Coot and automated refinement with Phenix refine (53). Geometric restraints for the substrate 8-oxo-rGTP were generated using the elbow program in Phenix (54). To confirm the presence and conformation of the 8-oxo-rGTP ligand, Fo-Fc omit map was calculated using Phenix polder map (55). The quality of the final models was validated using MolProbity (56). Atomic coordinates and structure factors for all four structures have been deposited in the Protein Data Bank (PDB) with accession codes 9PVU, 9PVV, 9PVW, and 9PVX. Data collection and refinement statistics are summarized in *SI Appendix*, Table S1.

### Structure-Based Stability Analysis Using FoldX.

To evaluate the thermodynamic stability of the syn-conformation versus the anticonformation of the 8-oxo-rG within the Pol II active site, we performed structure-based energy calculations using FoldX (57). Since the FoldX force field does not natively parameterize the 8-oxo-rG, it was modeled as a rG while preserving the base orientation (syn-, anti-). For energy calculation, the RepairPDB function was applied to both complexes to optimize side chain rotamers and resolve minor steric clashes while fixing the backbone. Subsequently, the AnalyseComplex function was used to calculate the interaction energy (ΔG) between 8-oxo-rG (defined as a separate chain Z) and the surrounding Pol II active site elements. We focused on the energy contributions from the Rpb2 subunit (Chain B, containing residue E529) and the nascent RNA 3’-end (Chain R). The interaction energy was compared between the syn- and anti-conformations.

## Supplementary Material

Appendix 01 (PDF)

## Data Availability

Structural data have been deposited in PDB (9PVU (29), 9PVV (30), 9PVW (31), and 9PVX (32)). All other data are included in the manuscript and/or *SI Appendix*.
