# Supplementary material for: Structural basis of transcription-coupled RNA damage by incorporation of oxidized ribonucleotides
Source: Proc Natl Acad Sci U S A. 2026 Apr 14;123(16):e2602266123. doi: 10.1073/pnas.2602266123 (PMC13099631; doi:10.1073/pnas.2602266123)
Supplement: Supplementary file 1 — Appendix 01 (PDF) [file pnas.2602266123.sapp.pdf]

Supplementary Information

Structural basis of transcription-coupled RNA damage by incorporation of oxidized ribonucleotides

Peini Hou[1], Chanjoo Lee[2,3,4], Jenny Chong[1], Juntaek Oh[2,3,4,*] Dong Wang[1,5,6,*]

[1] Department of Pharmaceutical Sciences, Skaggs School of Pharmacy and Pharmaceutical Sciences, University of California, San Diego, La Jolla, CA 92093

[2] Department of Regulatory Science, Graduate School, Kyung Hee University, Seoul, 02447, Republic of Korea

[3] Institute of Regulatory Innovation through Science, Kyung Hee University, Seoul, 02447, Republic of Korea

[4] Institute of Integrated Pharmaceutical Sciences, Kyung Hee University, Seoul, 02447, Republic of Korea

[5] Department of Cellular and Molecular Medicine, University of California, San Diego, La Jolla, California 92093, United States

[6] Department of Chemistry and Biochemistry, University of California, San Diego, La Jolla, California 92093, United States

[*] Corresponding author: dongwang@ucsd.edu, ojt@khu.ac.kr

This PDF file includes:

Figure S1-S4

Table S1-S7

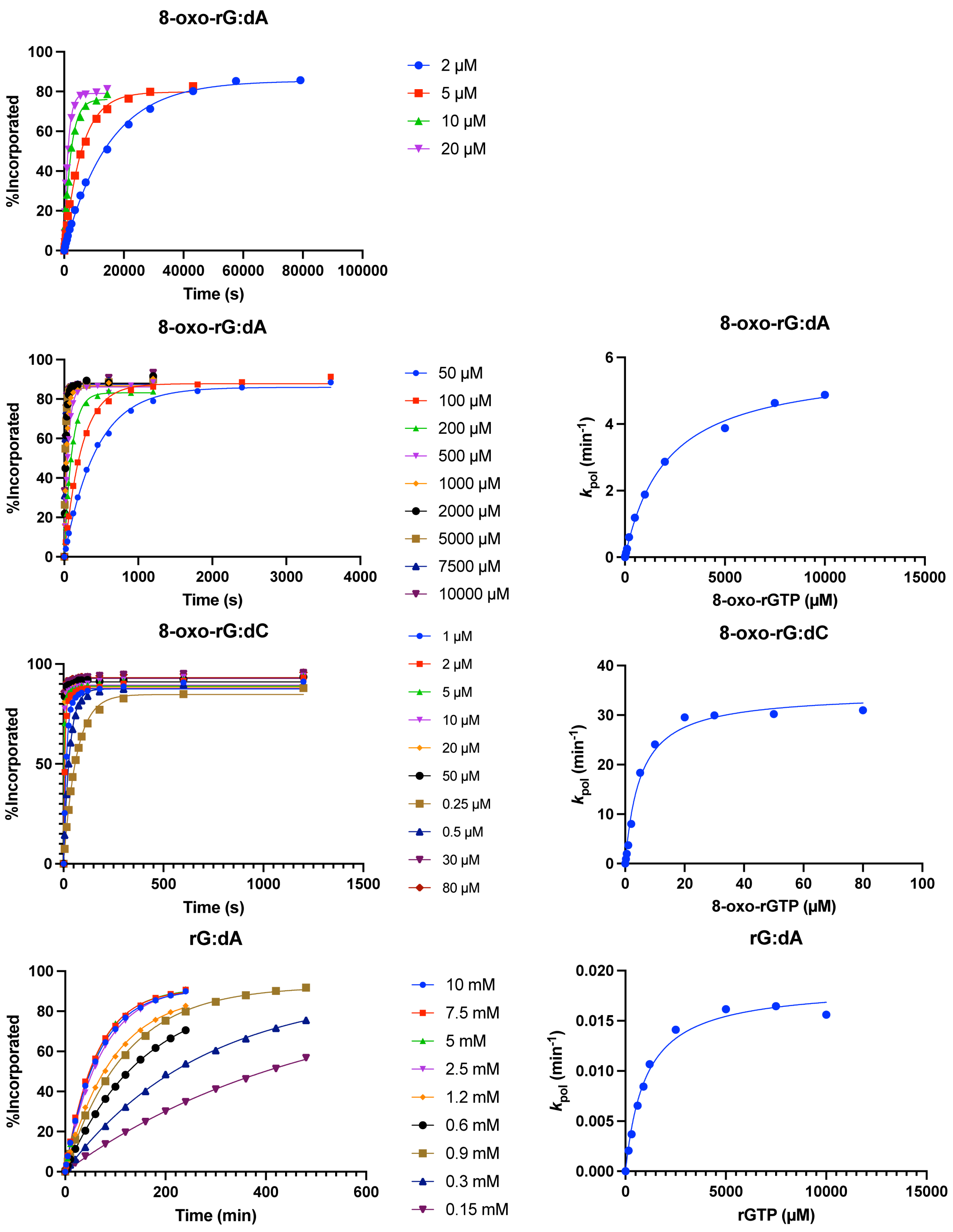

Figure S1. Single turn-over nucleotide incorporation kinetic analysis of 8-oxo-rG:dA, 8-oxo-rG:dC and rG:dA. Kinetic parameters were calculated from biochemical assays with incubation time ranging from 0 to several hours depending on the incorporation efficiency. The substrates and their concentrations for each assay are indicated in each corresponding plot. Regression curves were generated using Prism 10.

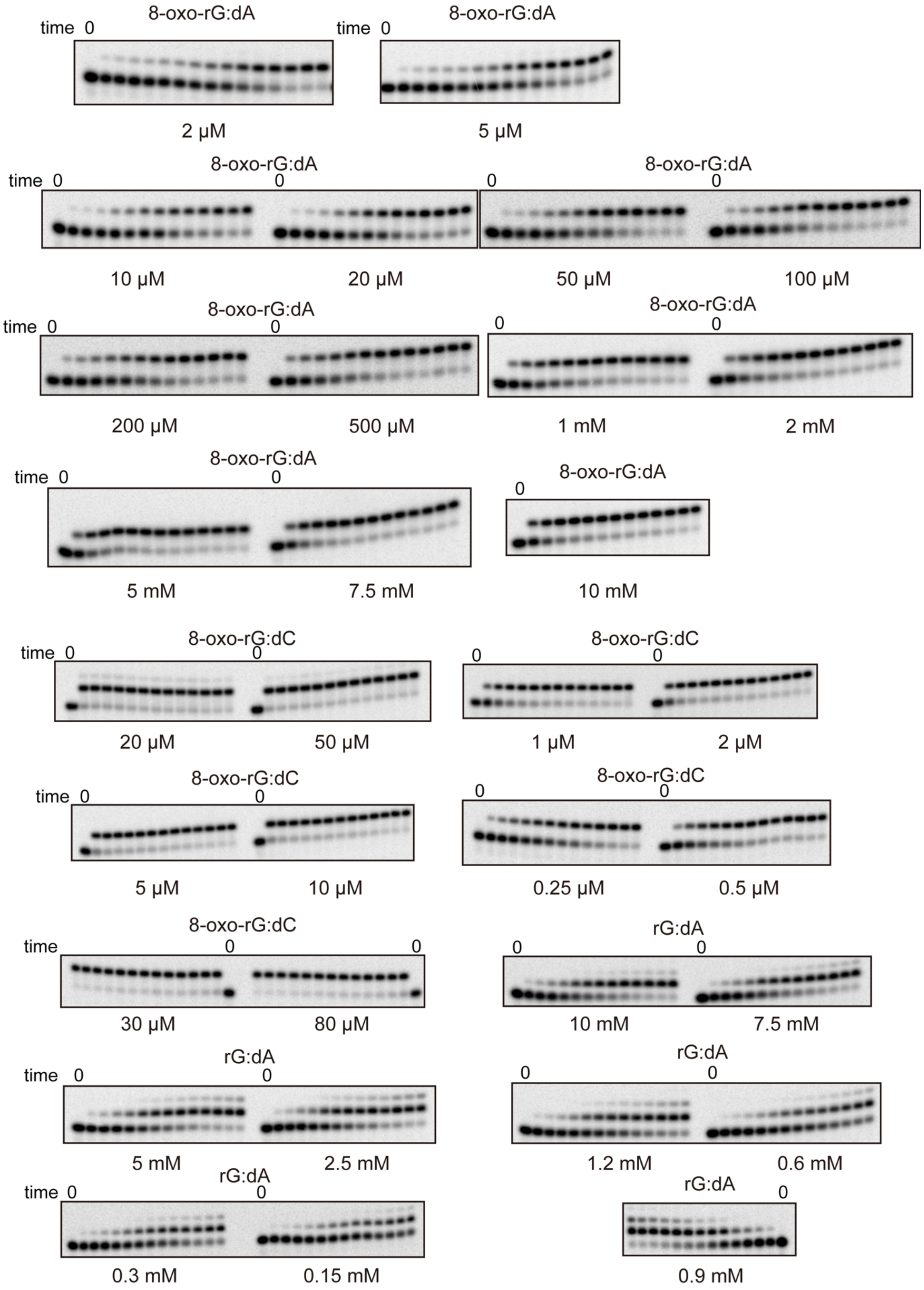

Figure S2. Raw gel data for single turn-over nucleotide incorporation assay.

Raw gel data for Figure S1. All samples were denatured by heating at 95°C for 10 minutes and subsequently subjected to analysis using a 12% denaturing urea-PAGE gel.

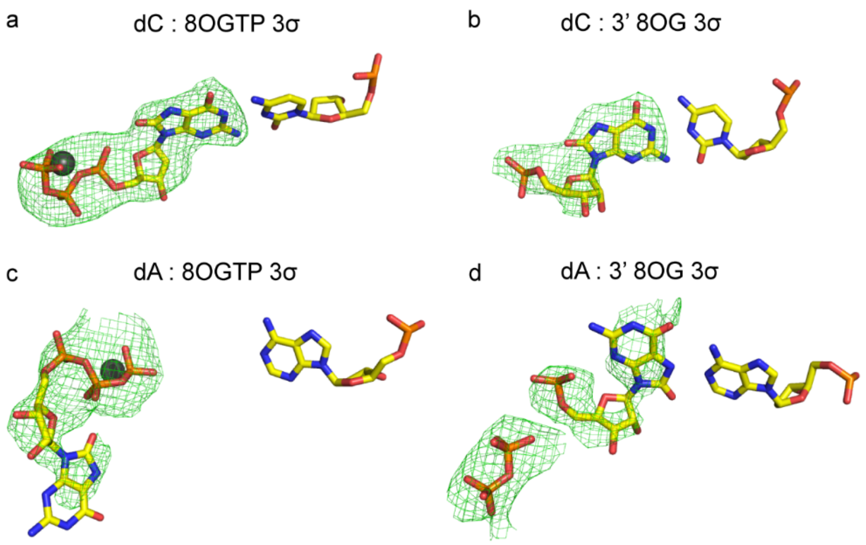

Figure S3. Fo-Fc omit density maps.

(a-d) Each panel displays 3σ-contoured Fo–Fc electron density map around 8OGTP and the template base, dC for (a) and (b) and dA for (c) and (d).

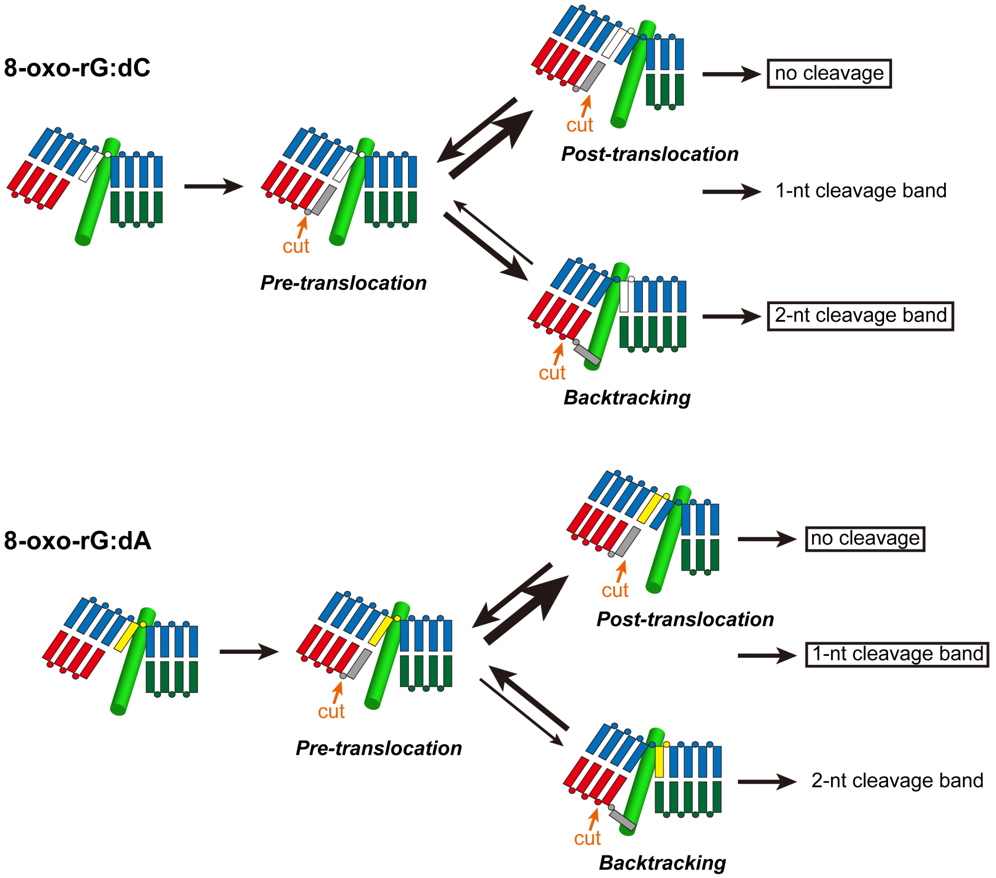

Figure S4. Schematic illustrating the characteristic 2-nt TFIIS-stimulated backtrack cleavage on dC template versus the 1-nt non-backtracked cleavage observed on dA template.

Table S1. Time points for single turn-over nucleotide incorporation kinetic analysis of 8-oxo-rG:dA.

8-oxo-rGTP concentration	Time points for 8-oxo-rG:dA
2 µM	0 s, 5 min, 10 min, 15 min, 20 min, 30 min, 40 min, 1 h, 1.5 h, 2 h, 4 h, 6 h, 8 h, 12 h, 16 h, 22 h
5 µM	0 s, 2 min, 5 min, 7.5 min, 10 min, 15 min, 20 min, 30 min, 1 h, 1.5 h, 2 h, 3 h, 4 h, 6 h, 8 h, 12 h
10 µM	0 s, 30 s, 1 min, 2 min, 5 min, 10 min, 15 min, 25 min, 40 min, 1 h, 1.5 h, 2 h, 3 h, 4 h
20 µM	0 s, 30 s, 1 min, 2 min, 5 min, 10 min, 15 min, 25 min, 40 min, 1 h, 1.5 h, 2 h, 3 h, 4 h
50 µM	0 s, 20 s, 40 s, 1 min, 2 min, 3 min, 5 min, 7.5 min, 10 min, 15 min, 20 min, 30 min, 40 min, 1 h
100 µM	0 s, 20 s, 40 s, 1 min, 2 min, 3 min, 5 min, 7.5 min, 10 min, 15 min, 20 min, 30 min, 40 min, 1 h
200 µM	0 s, 10 s, 20 s, 30 s, 45 s, 60 s, 1.5 min, 2 min, 3 min, 5 min, 7.5 min, 10 min, 15 min, 20 min
500 µM	0 s, 10 s, 20 s, 30 s, 45 s, 60 s, 1.5 min, 2 min, 3 min, 5 min, 7.5 min, 10 min, 15 min, 20 min
5 mM	0 s, 5 s, 15 s, 25 s, 35 s, 45 s, 60 s, 75 s, 90 s, 2 min, 3 min, 5 min, 10 min, 20 min
7.5 mM	0 s, 5 s, 15 s, 25 s, 35 s, 45 s, 60 s, 75 s, 90 s, 2 min, 3 min, 5 min, 10 min, 20 min
10 mM	0 s, 5 s, 15 s, 25 s, 35 s, 45 s, 60 s, 75 s, 90 s, 2 min, 3 min, 5 min, 10 min, 20 min

Table S2. Time points for single turn-over nucleotide incorporation kinetic analysis of 8-oxo-rG:dC.

8-oxo-rGTP concentration	Time points for 8-oxo-rG:dC
0.25 μM	0 s, 5 s, 15 s, 25 s, 35 s, 45 s, 60 s, 75 s, 90 s, 2 min, 3 min, 5 min, 10 min, 20 min
0.5 μM	0 s, 5 s, 15 s, 25 s, 35 s, 45 s, 60 s, 75 s, 90 s, 2 min, 3 min, 5 min, 10 min, 20 min
1 μM	0 s, 5 s, 15 s, 25 s, 35 s, 45 s, 60 s, 75 s, 90 s, 2 min, 3 min, 5 min, 10 min, 20 min
2 μM	0 s, 5 s, 15 s, 25 s, 35 s, 45 s, 60 s, 75 s, 90 s, 2 min, 3 min, 5 min, 10 min, 20 min
5 μM	0 s, 5 s, 15 s, 25 s, 35 s, 45 s, 60 s, 75 s, 90 s, 2 min, 3 min, 5 min, 10 min, 20 min
10 μM	0 s, 5 s, 15 s, 25 s, 35 s, 45 s, 60 s, 75 s, 90 s, 2 min, 3 min, 5 min, 10 min, 20 min
20 μM	0 s, 5 s, 15 s, 25 s, 35 s, 45 s, 60 s, 75 s, 90 s, 2 min, 3 min, 5 min, 10 min, 20 min
50 μM	0 s, 5 s, 15 s, 25 s, 35 s, 45 s, 60 s, 75 s, 90 s, 2 min, 3 min, 5 min, 10 min, 20 min
30 μM	0 s, 5 s, 15 s, 25 s, 35 s, 45 s, 60 s, 75 s, 90 s, 2 min, 3 min, 5 min, 10 min, 20 min
80 μM	0 s, 5 s, 15 s, 25 s, 35 s, 45 s, 60 s, 75 s, 90 s, 2 min, 3 min, 5 min, 10 min, 20 min

Table S3. Time points for single turn-over nucleotide incorporation kinetic analysis of rG:dA.

rGTP concentration	Time points for rG:dA
0.15 mM	0 s, 5 min, 10 min, 20 min, 40 min, 80 min, 120 min, 160 min, 200 min, 240 min, 300 min, 360 min, 420 min, 480 min
0.3 mM	0 s, 5 min, 10 min, 20 min, 40 min, 80 min, 120 min, 160 min, 200 min, 240 min, 300 min, 360 min, 420 min, 480 min
0.9 mM	0 s, 5 min, 10 min, 20 min, 40 min, 80 min, 120 min, 160 min, 200 min, 240 min, 300 min, 360 min, 420 min, 480 min
0.6 mM	0 s, 2 min, 5 min, 10 min, 20 min, 40 min, 60 min, 80 min, 100 min, 120 min, 150 min, 180 min, 210 min, 240 min
1.2 mM	0 s, 2 min, 5 min, 10 min, 20 min, 40 min, 60 min, 80 min, 100 min, 120 min, 150 min, 180 min, 210 min, 240 min
2.5 mM	0 s, 2 min, 5 min, 10 min, 20 min, 40 min, 60 min, 80 min, 100 min, 120 min, 150 min, 180 min, 210 min, 240 min
5 mM	0 s, 2 min, 5 min, 10 min, 20 min, 40 min, 60 min, 80 min, 100 min, 120 min, 150 min, 180 min, 210 min, 240 min
7.5 mM	0 s, 2 min, 5 min, 10 min, 20 min, 40 min, 60 min, 80 min, 100 min, 120 min, 150 min, 180 min, 210 min, 240 min
10 mM	0 s, 2 min, 5 min, 10 min, 20 min, 40 min, 60 min, 80 min, 100 min, 120 min, 150 min, 180 min, 210 min, 240 min

Table S4. Data collection and refinement statistics

	dC, 8-oxo-rGMP	dC, 3´dd RNA, 8-oxo-rGTP	dA, 8-oxo-rGMP	dA, 3´dd RNA, 8-oxo-rGTP
**PDB ID**	**9PVU**	**9PVV**	**9PVW**	**9PVX**
**Data collection**				
**Resolution, Å**	49.32 - 3.31 (3.43 - 3.31)[a]	38.38 - 3.48 (3.604 - 3.48)	48.75 - 3.56 (3.687 - 3.56)	38.44 - 3.39 (3.511 - 3.39)
**Space group**	C 1 2 1			
**Unit cell**				
**a b c**	166.0 223.5 192.8	159.5 222.7 191.2	166.0 223.2 192.8	159.7 222.3 192.1
**β (α, γ = 90°)**	100.3	97.8	100.5	97.9
**Unique reflections**	102682 (10193)	83659 (8315)	82438 (8185)	91011 (9135)
**Multiplicity**	2.0 (2.0)	2.0 (2.0)	2.0 (2.0)	2.0 (2.0)
**Completeness (%)**	99.6 (97.4)	98.7 (98.4)	99.7 (98.6)	99.0 (99.7)
**Mean I/sigma(I)**	5.27 (0.56)	5.59 (1.53)	4.70 (0.59)	6.55 (1.48)
**Wilson B-factor**	91.57	89.35	103.62	88.17
**R-merge**	0.19 (1.55)	0.083 (0.501)	0.22 (1.40)	0.076 (0.507)
**CC1/2[b]**	0.98 (0.25)[b]	1.00 (0.77)	0.98 (0.23)	1.00 (0.68)
**Refinement**				
**R-work / R-free**	0.243 / 0.281	0.218 / 0.258	0.247 / 288	0.192 / 0.236
**RMS (bonds)**	0.003	0.004	0.005	0.004
**RMS (angles)**	0.61	0.69	0.76	0.65
**Ramachandran outliers (%)**	0.03	0	0.03	0.03
**Rotamer outliers (%)**	0.23	0.23	0.29	0.2
**Clashscore**	16.8	18.7	26.8	13.0
**Average B-factor**	111.0	118.3	124.6	102.3

[a]Statistics for the highest-resolution shell are shown in parentheses.
[b]CC1/2 is used to define high resolution cutoff.

Table S5. Sequences of DNA and RNA oligos used in this study.

Oligo #	Sequences 5´ to 3´
1	AUCGAGAGG (RNA)
2	CCTTCTCTCTCTCGCTGGACCTCTCGATG (TS)
3	CCTTCTCTCTCTCGCTGGTCCTCTCGATG (TS)
4	CCTTCTCTCTCTCGCTGGGCCTCTCGATG (TS)
5	CCTTCTCTCTCTCGCTGGCCCTCTCGATG (TS)
6	CCAGCGAGAGAGAGAAGG (NTS)
7	CCAGCGAGAGAGAGAAGGTTTTTT-biotin
8	AUCGAGAGGA (RNA)
9	AUCGAGAGGC (RNA)
10	CCTTCTCTCTCTCGCTGA[8-oxoG]CCTCTCGATG (TS)
11	TCAGCGAGAGAGAGAAGG (NTS)
12	AUCGAGAGGG (RNA)

Table S6. Nucleic acid strand composition of elongation complex scaffolds.

figure	panel	Oligonucleotide composition
2	A	Incorporation scaffold 1: Oligo 2 (TS)+Oligo 6 (NTS)+Oligo 1 (RNA)
		Incorporation scaffold 2: Oligo 3 (TS)+Oligo 6 (NTS)+Oligo 1 (RNA)
		Incorporation scaffold 3: Oligo 4 (TS)+Oligo 6 (NTS)+Oligo 1 (RNA)
		Incorporation scaffold 4: Oligo 5 (TS)+Oligo 6 (NTS)+Oligo 1 (RNA)
3	A	Elongation scaffold 1: Oligo 2 (TS)+Oligo 7 (NTS)+Oligo 1 (RNA)
		Elongation scaffold 2: Oligo 5 (TS)+Oligo 7 (NTS)+Oligo 1 (RNA)
4	A	Proofreading scaffold 1: Oligo 10 (TS)+Oligo 11 (NTS)+Oligo 8 (RNA)
		Proofreading scaffold 2: Oligo 10 (TS)+Oligo 11 (NTS)+Oligo 9 (RNA)
		Proofreading scaffold 3: Oligo 2 (TS)+Oligo 7 (NTS)+Oligo 12 (RNA)
		Proofreading scaffold 4: Oligo 5 (TS)+Oligo 7 (NTS)+Oligo 12 (RNA)
		Proofreading scaffold 5: Oligo 2 (TS)+Oligo 7 (NTS)+Oligo 1 (RNA)
		Proofreading scaffold 6: Oligo 5 (TS)+Oligo 7 (NTS)+Oligo 1 (RNA)

Table S7. Interaction energy analysis of 8-oxo-rG in the Pol II active site.

Interaction	Energy Component	8OG (anti):dC	8OG (syn):dA	ΔΔG (Favoring)
Rpb2	Side-chain	-0.63*	-2.13**	-1.50 (syn)
RNA	Total Interaction	+0.39	-1.22	-1.61 (syn)
Template DNA	Total Interaction	-0.52	+0.11	+0.63 (anti)
Net Stabilization		-0.76	-3.24	-2.48 (syn)

*Negative values indicate stabilizing interactions. ΔΔG represents the energy difference, where negative values indicate greater stability for the syn-conformation.

**Values are in kcal/mol.
